# Stable Undifferentiated Connective Tissue Disease and CNS Demyelination: A Case Report and a Literature Review

**DOI:** 10.1155/crnm/3106627

**Published:** 2025-08-31

**Authors:** Andrew R. Pachner

**Affiliations:** Dartmouth-Hitchcock Medical Center and Geisel School of Medicine at Dartmouth, Lebanon, New Hampshire, USA

## Abstract

This patient had CNS demyelination in the context of stable undifferentiated connective tissue disease (sUCTD) and a relatively benign course despite minimal treatment. Her course is consistent with the course of similar patients described in the literature, which supports a relatively conservative approach to treatment in this group of patients.

## 1. Introduction

This 17-year-old woman presented in 2010 with an episode of Kikuchi's necrotizing cervical lymphadenitis confirmed by biopsy. She had a second, third, and fourth episode in 2013, 2015, and 2021, and after 2013, she subsequently also had an episode of pericarditis and nonspecific arthralgias not fulfilling EULAR/ACR criteria for joint involvement in SLE [1]. She developed optic neuritis in 2015, was evaluated for multiple sclerosis (MS), and was found to have MRI brain lesions and positive oligoclonal bands in her cerebrospinal fluid (CSF) consistent with MS, and she was started on glatiramer acetate, an MS disease-modifying therapy, which she only took for less than a year. Over the next two years, she developed a few more brain white matter MRI lesions, but none since 2017, and over the course of 2015–2025, she never developed more clinical or imaging sequelae of CNS demyelination despite the fact that she has been off of all MS disease-modifying therapies since 2018. She has been treated for worsening symptoms of undifferentiated connective tissue disease (UCTD), usually of lymphadenitis, with only very short courses because of insurance problems: 2 courses of rituximab (9/2018 and 3/2018), two 3-day courses of methylprednisone, and 2 very short courses of belimumab. Laboratory evaluation has included the following: chronically elevated CRP and positive ANA speckled and negative testing for the following: anti-dsDNA, -Ro, -La, -Sm, and -RNP. ESR has been intermittently elevated, at one point being associated with inflammatory arthritis. The two rheumatologists caring for her during the course of her illness felt that she did not meet criteria for the diagnosis of SLE. Tests for antibodies to aquaporin-4 (AqP4) and myelin oligodendrocyte glycoprotein (MOG) were also negative. In 2025, her neurological examination was completely normal, and her imaging revealed only a few small FLAIR hyperintensities on MRI brain and a small white matter lesion at T11 on cord imaging.

The patient gave consent to publish this information.

## 2. Discussion

UCTD can be defined as the presence of clinical symptoms of a systemic autoimmune disease in addition to laboratory evidence of autoimmunity with the patients not fulfilling any of the widely used classification criteria for classic autoimmune diseases. UCTD can be further be subcategorized into evolving (eUCTD), usually early in the course, or stable UCTD (sUCTD), based on whether or not it evolves into a defined rheumatological disease [2]. The distinction can have clinical importance with regard to monitoring and treatment. The connective tissue disease thought by this patient's clinicians to be most consistent with her presentation was systemic lupus erythematosus(SLE), but since she did not fulfill diagnostic criteria for SLE (1), she was diagnosed as UCTD, which by 15 years after its onset was further defined as sUCTD.

There is an extensive literature on neuropsychiatric manifestations of SLE, but within these presentations, demyelinating syndromes are rare [3], and when they occur, they tend to have a benign course [4]. Moreover, MS can rarely coexist with SLE, but when it does neither the SLE nor the MS has a severe phenotype [5]. CNS demyelinating syndromes in UCTD are even rarer. Radin et al. [6] found no neurological complications in 104 patients with UCTD followed over an average of 6.2 years. In the very rare patients with UCTD and CNS demyelination, usually reported in case reports, the CNS demyelination is in the form of myelitis (“lupus myelitis”), or has been part of neuromyelitis optica (NMO) or MOG-associated disease (MOGAD) [7, 8]; the patient in this report had negative aquaporin-4 and MOG antibodies and did not have a clinical course consistent with NMO or MOGAD.

She fulfilled McDonald 2017 diagnostic criteria for MS when she developed optic neuritis and underwent a lumbar puncture and imaging. However, her neurological clinical course was very mild despite minimal treatment. When her course is assessed using the Multiple Sclerosis Severity Score (MSSS) [9], her score is less than 0.19 on a scale of 0–10, indicating much less severe disability accrual over time than the norm in MS. The course of MS and UCTD can be modified by sustained immunosuppression, but the benignity of this patient's course is unlikely to be explained by medication effect, since she was on immunosuppression for less than 10% of the course of her disease.

The absence of progression of sUCTD to a defined rheumatological disease may indicate that downregulatory mechanisms, such as elevated type I interferons and IL-10 [10], are potent in these patients, possibly explaining why the neuroinflammation in this patient has been relatively mild.

This case report, along with the associated literature cited, is instructive because it assists neurologists, rheumatologists, and other clinicians in managing patients with UCTD and CNS demyelination. These patients, such as the patient described in this report, would be predicted to have relatively mild demyelinating syndromes with excellent prognoses over time. Thus, benefit/risk calculations in these patients might favor less aggressive, and less risky, therapies of their demyelination.

## Data Availability

The data that support the findings of this study are available on request from the corresponding author. The data are not publicly available due to privacy or ethical restrictions.

## References

[1] Aringer M., Costenbader K., Daikh D. (2019). 2019 European League Against Rheumatism/American College of Rheumatology Classification Criteria for Systemic Lupus Erythematosus. *Arthritis and Rheumatology*.

[2] Rubio J., Kyttaris V. C. (2023). Undifferentiated Connective Tissue Disease: Comprehensive Review. *Current Rheumatology Reports*.

[3] Jayasinghe M., Rashidi F., Gadelmawla A. F. (2025). Neurological Manifestations of Systemic Lupus Erythematosus: A Comprehensive Review. *Cureus*.

[4] Nikolopoulos D., Kitsos D., Papathanasiou M. (2022). Demyelinating Syndromes in Systemic Lupus Erythematosus: Data from the Attikon Lupus Cohort. *Frontiers in Neurology*.

[5] Fanouriakis A., Mastorodemos V., Pamfil C. (2014). Coexistence of Systemic Lupus Erythematosus and Multiple Sclerosis: Prevalence, Clinical Characteristics, and Natural History. *Seminars in Arthritis and Rheumatism*.

[6] Radin M., Rubini E., Cecchi I. (2022). Disease Evolution in a Long-Term Follow-Up of 104 Undifferentiated Connective Tissue Disease Patients. *Pathogenesis of Rheumatoid Arthritis: One Year in Review 2022*.

[7] Krett J. D., Filippatou A. G., Barreras P., Pardo C. A., Gelber A. C., Sotirchos E. S. (2025). Lupus Myelitis Revisited: A Retrospective Single-Center Study of Myelitis Associated With Rheumatologic Disease. *Neurology Neuroimmunology and Neuroinflammation*.

[8] Protti A., Erminio C., Piccolo I., Spreafico C., Colombo F., Ghezzi A. (2004). An Unusual Case With Relapsing Neuromyelitis Optica Associated With Undifferentiated Connective Tissue Disease. *Neurological Sciences*.

[9] Roxburgh R. H., Seaman S. R., Masterman T. (2005). Multiple Sclerosis Severity Score: Using Disability and Disease Duration to Rate Disease Severity. *Neurology*.

[10] Kim S. T., Munoz-Grajales C., Dunn S. E. (2023). Interferon and Interferon-Induced Cytokines as Markers of Impending Clinical Progression in ANA (+) Individuals Without a Systemic Autoimmune Rheumatic Disease Diagnosis. *Arthritis Research and Therapy*.

